# Modulatory effect of dietary probiotic and prebiotic supplementation on growth, immuno-biochemical alterations, DNA damage, and pathological changes in *E. coli*-infected broiler chicks

**DOI:** 10.3389/fvets.2022.964738

**Published:** 2022-10-20

**Authors:** Mohamed A. Hashem, Azza E. A. Hassan, Hala M. M. Abou-Elnaga, Walied Abdo, Naief Dahran, Ali H. Alghamdi, Ehab Kotb Elmahallawy

**Affiliations:** ^1^Department of Clinical Pathology, Faculty of Veterinary Medicine, Zagazig University, Zagazig, Egypt; ^2^Department of Biochemistry, Animal Health Institute, Mansoura, Egypt; ^3^Department of Clinical Pathology, Animal Health Institute, Mansoura, Egypt; ^4^Department of Pathology, Faculty of Veterinary Medicine, Kafrelsheikh University, Kafr El-Sheikh, Egypt; ^5^Department of Anatomy, Faculty of Medicine, University of Jeddah, Jeddah, Saudi Arabia; ^6^Department of Biology, Faculty of Science, Albaha University, Al Aqiq, Saudi Arabia; ^7^Department of Zoonoses, Faculty of Veterinary Medicine, Sohag University, Sohag, Egypt

**Keywords:** modulatory, probiotic, prebiotic, immuno-biochemical alteration, DNA, histopathological, *E. coli*, broilers

## Abstract

Avian pathogenic *Escherichia coli* is one of the principal causes of heavy economic losses to the poultry industry. Little is known about the underlying mechanisms, particularly the potential role of immunoglobulin A and the DNA damage, involving the beneficial effects of dietary supplementation of probiotics and prebiotics in avian colibacillosis. The current study investigated the potential effects of probiotic and prebiotic dietary supplementation on *E. coli*-infected broiler chicks. A total of 120 1-day-old unsexed Hubbard chicks were divided into six groups: Group 1 was considered as a negative control; Group 2 was supplemented with 1 g/kg feed of *Lactobacillus plantarum*; Group 3 was supplemented with amylase enzyme; Group 4 served as a positive control infected orally by *E. coli* O78; Group 5 was supplemented with *L. plantarum* from 1-day-old chicken and then infected orally with *E. coli* O78; and Group 6 was supplemented with amylase enzyme from 1-day old chicken and then infected orally with *E. coli* O78. For all examined groups, the experimental period lasted for 42 days. The *E. coli-infected* group revealed a decrease in body performance parameters with a significant increase in the liver enzymes and renal function tests. The same group recorded a significant decrease in serum total proteins, albumins, and globulins, and the alteration of immunological parameters, antioxidant enzymes, oxidative stress parameters, and comet assay revealed highly damaged DNA in the liver and the intestine. By histopathological examination, a series of histopathological changes in the liver, the kidney, and the intestine were observed. The infected chick pretreated with probiotics or prebiotics demonstrated an improvement in body performance parameters besides a significant decrease in the hepatic enzymes and renal function tests. We noticed that, in treated groups, there was a significant increase in serum total proteins in the serum albumin and globulin levels, immunological parameters, and antioxidant enzymes. Furthermore, DNA damage and histopathological changes within hepatic, renal, and intestinal tissues were markedly diminished in the treated groups compared with the infected group. We concluded that the adverse effects of *E. coli* could be modulated through the chemopreventive administration of probiotics and prebiotics.

## Introduction

Avian colibacillosis is considered one of the most important bacterial diseases that target the poultry industry, resulting in severe economic losses in broilers (Kabir, 2010). The disease is considered one of the principal causes of morbidity and mortality in broilers that reach up to 50%, either as a primary or as a secondary pathogen (Kabir, 2010). *Escherichia coli* (*E. coli*) causes avian colibacillosis, which is considered a natural commensal inhabitant of the chicken's intestinal tract and the trachea to a lesser extent. Commonly, 10–15% of intestinal *E. coli* are avian pathogenic *E. coli* (APEC) with various virulence factors, which might result in systemic diseases such as pericarditis, perihepatitis, airsaculities, and peritonitis (1). A total of 20 *E. coli* serotype isolates were recovered in poultry, including O6, O111, O55, O114, O15, O125, and O78 (2). Infection is stimulated when a bird's defense mechanism falls, potentiated by various factors such as bad management, concurrent infections, and immunosuppression (3). No effective vaccine is available against colibacillosis, mainly as a result of the variety of APEC strains existing in the field. Therefore, prophylactic measures and antibiotic treatment are required to control colibacillosis. Antibiotic use is considered the most common way for treating colibacillosis. This strategy might possess major side effects on the bird and consumers combined with the development of drug resistance.

Feed additives are an important strategy. They have been used to advance the efficiency of poultry industry and the performance and health of animals by targeting growth efficiency, disease prevention, and feed utilization improvement (4). Some studies revealed that feed additives have no harmful effects on human and animal health and on the environment (4). Probiotics are live categories of feed additives that consist of one single strain or a combination of several strains of non-pathogenic microorganisms such as bacteria (i.e., *Enterococcus, Bacillus, Lactobacillus plantarum*, and *Pediococcus*), yeast, and fungi, which are probiotics, which beneficially improve the gut microflora of the host (5). Among others, *L. plantarum* is an antimicrobial feed additive probiotic with interesting activity against various poultry pathogens since they inhibit the progress of *E. coli*, enhance growth performance, reduce *Enterobacteriaceae* population, and increase *Lactobacillus* numbers in broilers (6). The probiotic mode of action is mainly based on competitive exclusion, bacterial antagonism, and immune modulation (7). Fermentable sugars (i.e., fructo-oligo-saccharides and galacto-oligo-saccharides) and exogenous enzymes (8) are prebiotics that are non-digestible food ingredients that are used as feed additives to improve host health and protect poultry against pathogens. Amylase, xylanase, and protease are exogenous enzymes, which are a group of proteins that facilitate specific chemical reactions, increase digestion, and are, therefore, supplemented to animal diet (9). It should be highlighted that the amylase enzyme is the exogenous bacterial enzyme commonly added to a bird's diet to match the requirement of birds and improve its performance by increasing nutrient utilization and maintaining health status (10). The mode of action of exogenous enzymes mainly targets lower gut viscosity, leading to complete digestion and absorption of nutrients, reduced microbial proliferation, and improved gut health by selectively stimulating the growth and/or activity of one or a limited number of bacteria (11). From the available literature, several previous studies investigated the potential benefits of dietary probiotic and prebiotic supplementations on animal and poultry diets (12–14). Little information is available about the mechanistic pathways underlying these potential beneficial effects of these supplements in the poultry industry and the potential role of immunoglobulins A and DNA damage using the comet assay test. The present study is aimed at understanding how these feed additives can be used to control *E. coli* infection and thus improving the efficiency of poultry production.

## Materials and methods

### Ethical approval

The animal care and experimental protocols were approved by the ZU-IACUC committee, Faculty of Veterinary Medicine, Zagazig University, Egypt. The approval number of the study is ZU-IACUC/2/F/88/2021.

### Tested compounds

*Lactobacillus plantarum* (1.6 Billion CFU) was purchased as a biotechnology grade probiotic bacterial powder from the Egyptian Company for Biotechnological, Cairo, Egypt. Meanwhile, amylase enzyme was also purchased as a biotechnology grade exogenous prebiotic enzyme product produced from natural anaerobic bacteria from New Vet Company, Mansoura, Egypt.

### Bacteria (the inoculum's bacteria) (*E. Coli* strain)

In the present study, a virulent *E. coli* strain, serotype O78, was kindly supplied, identified, classified, and serotyped by the Microbiology Department, Zagazig University as described in a previous study (15). The used strain of *E. coli* was first demonstrated to be pathogenic in a preliminary infectivity trial (pilot experiment) (16). The *E. coli* inoculum was a logarithmic phase culture produced by overnight incubation of *E. coli* bacteria in a nutrient broth (17). The number of bacteria per milliliter was determined by plating 10-fold serial dilution of the nutrient broth suspension on plate count agar. Titers were expressed as colony-forming units per ml (CFU/ml) as described in a previous study (18). Approximately, 1 ml of inoculum (broth culture) containing 3 × 10^7^ viable CFU of *E. coli* strain O78/1 ml/chicks were experimentally infected to the chicks orally *via* intra-crop (19).

### Animals and experimental design

The experimental treatments and sampling timeline are presented in Table 1. A total of 120 1-day-old unsexed chicks of Hubbard strain broilers were obtained from a hatchery in Dakahlia Poultry Company, Dakahlia, Egypt. The experiment lasted for 42 days with good ventilation. Birds were randomly divided into six groups (20 each) and raised in an open, well-ventilated house with sawdust. Rearing was initiated in floor pens until 7 days old following all hygienic measures. Chickens were then moved to wire-floored cages until the end of the experiment. Tap water and a balanced commercial ration free from antibiotics and anticoccidial were provided to the chickens *ad libitum*, and its ingredients and chemical compositions were formulated as presented elsewhere (20). The optimum temperature was adjusted using electric radiators and ventilators, which were set at 34°C during the first week and gradually reduced by 3°C every week until it reached 24°C. In addition, the light program for the first week was 24 h a day and then changed to 16 h of light and 8 h of dark over 7 to 42 days. All birds were vaccinated against Newcastle disease at 7 and 14 days old and 11 and 22 days old for Gumboro disease (21).

**Table 1:** The timeline of the experimental protocol explaining the day (D) number, treatments, and sampling.

**Groups**	**Number of chicks**	**Treatments (type, route, and duration)**	**Time of sampling**
G1 (control)	20	Control normal ration for 42 days.	Samples were collected at 23rd and 37th days old
G2 (*L. plantarum*)	20	*Lactobacillus Plantarum* (1.6 × 10^12^ CFU) (1 g/kg feed) for 42 days.	
G3 (amylase enzyme)	20	Amylase enzyme (0.6 g/kg feed) for 42 days.	
G4 (infection)	20	Infected with *E. coli* O78 (3 × 10^7^) at 10th day old (1 ml /bird orally intra-crop) for 42 days.	
G5 (*L. plantarum* + infection)	20	*Lactobacillus plantarum* (1.6 × 10^12^ CFU) (1 g/kg feed) and infected with *E.coli* O78 (3 × 10^7^) at 10th day (1 ml *E. coli*/ bird orally intra-crop) for 42 days.	
G6 (amylase enzyme + infection)	20	Amylase enzyme (0.6 g/kg feed) and infected with *E.coli* O78 (3 × 10^7^) at 10th day (1 ml *E. coli*/bird orally intra-crop) for 42 days.	

### Growth performance

The average initial body weight (BW) was reported at the beginning of the experiment. BW was then calculated every week as described in a previous study (22), and body weight gain (BWG) was determined (23). The difference between the weight of the provided feed and the feed that remained was used to calculate feed intake (FI) per replicate. The feed conversion ratio (FCR) was estimated according to the following equation (24):


$$\begin{array}{l} FCR=amount\;of\;consumed\;feed\;\left(g\right)/BWG\;\left(g\right) \end{array}$$

### Blood sampling and tissue collection

Blood samples were drawn from the wing vein of each group on the 23rd and 37th day from the start of the experiment. Approximately, 5 ml of blood was used to separate the serum samples, which were then kept at −20°C for further biochemical, immunological, and antioxidant analyses. Meanwhile, 2 ml of blood taken was collected in a heparinized tube for phagocytic activity and phagocytic index test. On the 23rd and 37th days of the experiment, chickens were killed by neck dislocation, specimens from both the liver and the intestine were collected, placed in an ice-cold phosphate buffer saline (BPS), and kept at −20°C until further use for the comet assay. Specimens from the liver, the kidney, and the intestine were collected, fixed in a 10% neutral buffered formalin solution, and trimmed for histopathological examination.

### Serum biochemical parameters

The serum activities of alanine aminotransferase (ALT) and aspartate aminotransferase (AST) were determined (25). Serum alkaline phosphatase activity (ALP) and urea were also measured (26), and the serum creatinine levels were determined according to Henry (27). Serum uric acid was estimated according to Fossati et al. (28). The serum total protein and albumin levels were evaluated according to Grant (29) and Doumas et al. (30), respectively. The serum globulin levels were calculated mathematically by subtracting albumin values from total protein values, as described in a previous study (31). The phagocytic activity and phagocytic index test were estimated according to Wilkinson (32), while serum immunoglobulin A (IgA) was estimated according to Bianchi et al. (33). The serum interleukin-6 level was determined according to Chan and Perlstein (34). Furthermore, serum malondialdehyde (MDA), superoxide dismutase (SOD), and catalase (CAT) were determined (35–37).

### *Comet* assay (single cell gel electrophoresis)

The detection of DNA damage was measured in the liver and the intestinal tissues using comet assay (38). Tissues were embedded in 0.6% normal melting point and low melting point agarose gel on microscope slides. The slides were then immersed in lysing solution (2.5 mol NaCl, 100 Mm Na_2_EDTA, freshly added 1%Triton-x100, and 10% DMSO) for 1 h at 4°C for denaturation and unwinding for DNA. Slides were placed in an electrophoresis buffer (300 mM NaOH, 1 mM Na2EDTA PH. 13.0) at 4°C for 30 min. Neutralization was performed using Tris-Hcl buffer (400 Mm Tris-Hcl, PH 7.4) followed by final staining with a fluorescent dye (20 ug/ml ethidium bromide). Observation of the DNA damage was determined by measuring the tail length and tail moment using X 40 objective on a fluorescent microscope (Nikon Microscope-Eclipse, E600 with Y-FL EPI-Fluorescence attachment, Japan) equipped with an excitation filter of 515–560 nm, barrier filter of 590 nm, and automatic digital imaging system running Comet assay TM software (perceptive Instrument, UK).

### Histopathological examination

Specimens from internal organs (mainly the liver, the kidney, and the intestine) were collected on the 23rd and 37th days from all groups and fixed in a 10% neutral buffered formalin solution, dehydrated in graded ethanol (70–100 %), cleared in xylene, and implanted in paraffin. A total of 5 μm thick paraffin sections were prepared and regularly stained with hematoxylin and eosin (HE) dyes and then examined using a standard light microscope (39).

### Statistical analysis

Data were analyzed by the analysis of variance method (ANOVA) using SPSS 18.0 software (40). Duncan multiple range tests were used to compare the means and the significance of differences which is considered at *p* ≤ 0.05.

## Results

### Clinical Signs and body performance

The experimentally *E. coli-infected* non-treated chicks exhibited ruffled feathers, inability to stand, dropping wings, sunken eyes, in-appetence, dullness, depression, decreased BW, breathing difficulty and gasping, sneezing and coughing, beak fluid discharge, white to yellowish diarrhea, and high mortality rate, which reached 35% and appeared 5–7 days post infection. Taking this into account, the severity of clinical signs was decreased in the experimentally *E. coli-infected* chicks treated with *L. plantarum* and amylase enzyme, whereas some chicks exhibited decreased appetite, decreased BW, respiratory rales, sneezing, and a lower mortality rate of 10–15%. As illustrated in Table 2, *E. coli*-infected non-treated chicks experienced a significant decrease in BW, BWG, and FI when compared with the control group. However, a highly significant increase in the BW, BWG, and FI were detected in birds treated with *L. plantarum* and amylase enzyme as compared to the normal control group (*p* < 0.05). These parameters were improved in the *E. coli*-infected treated groups with *L. plantarum* and amylase enzyme as compared to the *E. coli*-infected non-treated group during the experimental period. The weekly FCR exhibited a highly significant decrease in the groups treated with *L. plantarum* and amylase enzyme during the experimental period. A significant increase was noticed in the group infected with *E. coli*, and the non-treated compared with the control group. In addition, the groups infected with *E. coli* and treated with *L. plantarum* and amylase enzyme showed a significant improvement in the FCR as compared to the *E. coli-infected* non-treated group.

**Table 2:** Effect of probiotic and prebiotic supplementation on the growth performance in chickens of different groups (mean values ± S.E).

**Parameters**	**Weeks**	**Control**	***Lactobacillus plantarum***	**Amylase**	***E.coli***	***Lactobacillus plantarum*** **+ *E. coli***	**Amylase + *E. coli***	**P value**
BW (g/bird)	Int. BW	44.9 ± 0.66	44.9 ± 0.66	44.9 ± 0.066	44.9 ± 0.66	44.9 ± 0.66	44.9 ± 0.66	1.000
	1st week	155 ± 3.16	159 ± 7.48	158 ± 6.04	155 ± 3.16	159 ± 7.48	158 ± 6.04	0.211
	2ndweek	439 ± 9.27^b^	505 ± 9.74^a^	498 ± 7.07^a^	347 ± 8.60^c^	370 ± 7.07^c^	375 ± 4.47^c^	< 0.0001
	3rd week	849 ± 15.84^b^	994 ± 15.03^a^	980 ± 9.87^a^	677 ± 22.22^d^	746 ± 12.08^c^	750 ± 14.14^c^	0.001
	4th week	1323 ± 25.07^b^	1520 ± 25.69^a^	1508 ± 12.88^a^	1066 ± 27.49^d^	1190 ± 7.74^c^	1192 ± 22.89^c^	< 0.0001
	5th week	1848 ± 44.98^b^	2116 ± 33.25^a^	2106 ± 24.56^a^	1496 ± 14.35^d^	1684 ± 22.49^c^	1688 ± 13.56^c^	0.001
	6th week	2396 ± 67.94^b^	2746 ± 35.58^a^	2735 ± 20.12^a^	1933 ± 18.41^d^	2190 ± 48.47^c^	2208 ± 30.23^c^	< 0.0001
BWG (g/bird)	1st week	110.1 ± 5.43	115.1 ± 2.18	113.1 ± 6.58	110.1 ± 5.43	115.1 ± 2.18	113.1 ± 6.58	0.967
	2ndweek	284 ± 2.91^b^	345 ± 5.00^a^	340 ± 11.83^a^	192 ± 3.74^d^	211 ± 6.40^c^	217 ± 3.74^c^	0.001
	3rd week	410 ± 8.94^b^	489 ± 5.09^a^	482 ± 9.69^a^	330 ± 11.40^d^	376 ± 9.27^c^	375 ± 8.94^c^	< 0.0001
	4th week	474 ± 6.96^b^	526 ± 6.59^a^	528 ± 10.67^a^	389 ± 8.42^d^	444 ± 15.92^c^	442 ± 8.60^c^	< 0.0001
	5th week	525 ± 7.07^b^	596 ± 5.09^a^	598 ± 8.60^a^	430 ± 7.07^d^	494 ± 4.84^c^	496 ± 6.00^c^	0.001
	6th week	548 ± 7.34^b^	630 ± 8.36^a^	629 ± 6.40^a^	437 ± 10.19^d^	506 ± 6.00^c^	520 ± 7.07^c^	0.001
Feed intake (g/bird)	1st week	135 ± 3.53	140 ± 2.23	136 ± 4.30	135 ± 3.53	140 ± 2.23	136 ± 4.30	0.994
	2ndweek	381 ± 6.78^b^	421 ± 7.81^a^	413 ± 3.74^a^	287 ± 6.24^c^	290 ± 7.04^c^	296 ± 9.27^c^	0.007
	3rd week	632 ± 5.83^b^	670 ± 8.94^a^	672 ± 6.63^a^	570 ± 5.24^d^	592 ± 9.30^c^	594 ± 7.64^c^	< 0.0001
	4th week	830 ± 7.07^b^	854 ± 7.58^a^	852 ± 9.16^a^	754 ± 9.79^d^	790 ± 8.36^c^	796 ± 7.48^c^	< 0.0001
	5th week	940 ± 10.0^b^	983 ± 4.35^a^	980 ± 7.07^a^	850 ± 7.07^d^	896 ± 7.48^c^	898 ± 14.62^c^	0.001
	6th week	1030 ± 9.48^b^	1090 ± 13.78^a^	1070 ± 20.49^a^	937 ± 15.93^d^	985 ± 6.32^c^	990 ± 10.95^c^	< 0.0001
FCR	1st week	1.23 ± 0.06	1.21 ± 0.05	1.20 ± 0.03	1.23 ± 0.06	1.21 ± 0.05	1.20 ± 0.03	0.986
	2ndweek	1.34 ± 0.01^b^	1.22 ± 0.02^c^	1.21 ± 0.02^c^	1.49 ± 0.05^a^	1.37 ± 0.03^b^	1.36 ± 0.05^b^	0.009
	3rd week	1.54 ± 0.07^b^	1.37 ± 0.03^c^	1.39 ± 0.03^c^	1.73 ± 0.03^a^	1.57 ± 0.03^b^	1.58 ± 0.04^b^	0.005
	4th week	1.75 ± 0.03^b^	1.62 ± 0.03^c^	1.61 ± 0.03^c^	1.94 ± 0.04^a^	1.77 ± 0.06^b^	1.80 ± 0.04^b^	0.001
	5th week	1.79 ± 0.03^b^	1.64 ± 0.04^c^	1.63 ± 0.06^c^	1.97 ± 0.03^a^	1.81 ± 0.04^b^	1.81 ± 0.06^b^	< 0.0001
	6th week	1.88 ± 0.05^b^	1.73 ± 0.03^c^	1.70 ± 0.03^c^	2.14 ± 0.05^a^	1.94 ± 0.02^b^	1.90 ± 0.04^b^	< 0.0001

a, b, c, d Means carrying different superscripts are significantly different at p < 0.05. BW, body weight; BWG, body weight gain; FCR, feed conversion ratio.

### Effects of probiotic and prebiotic supplementation on some biochemical parameters

As shown in Table 3, the administration of *L. plantarum* or amylase enzyme reported a significant decrease in serum liver enzymes (ALT, AST, and ALP) and in the parameters of the kidney function test (urea, creatinine, and uric acid) at both periods of the experiment (23rd and 37th days of experiment) as compared to the control group. The *E. coli* infected non-treated chicks revealed a significant increase in the serum liver enzymes level and in the parameters of the kidney function tests when compared with the normal control group. Also, the treatment of infected chicks with either *L. plantarum* or amylase enzyme revealed an improvement in serum liver enzymes and the kidney function tests, which showed a significant decrease in G4, and the lowest value was reported in G5, which then returned near to normal at the end of the experimental period. Regarding serum total protein and albumin, birds treated with *L. plantarum* or amylase enzyme showed a significant increase in their values. However, a significant decrease in serum total protein and albumin values was noticed in *E. coli-infected* non-treated group when compared with the control group. On the contrary, the infected *E. coli* birds treated with *L. plantarum* or amylase enzyme recorded an improvement in serum total protein and albumin in comparison with the *E. coli-infected* non-treated birds over the experimental period. Regarding the serum total globulin, a significant increase was recorded in all groups, and this increase was more pronounced in G4 and less pronounced in G2 and G3 on the 23rd day of the experiment as compared to G1. On the contrary, on the 37th day of the experiment, the treated groups with *L. plantarum* or amylase enzyme showed a significant increase in serum total globulin. A significant decrease was noticed in the *E. coli*-infected non-treated group as compared to the control group. Compared with the *E. coli-infected* non-treated group, G5 and G6 showed an improvement expressed by a significant increase in the serum total globulin and returned to near-normal values at the end of the experiment.

**Table 3:** Effect of probiotic and prebiotic supplementation on some biochemical parameters in chickens of different groups at 23th and 37th day of the experimental period (mean values ± S.E).

**Parameters**	**Period**	**Control**	***Lactobacillus plantarum***	**Amylase**	***E. coli***	***Lactobacillus plantarum*** **+ *E. coli***	**Amylase + *E.coli***	**P value**
ALT (U/L)	23th day	9.10 ± 0.14^d^	7.30 ± 0.21^e^	7.50 ± 0.21^e^	12.90 ± 0.42^a^	10.48 ± 0.19^c^	11.30 ± 0.27^b^	< 0.0001
	37th day	10.64 ± 0.35^d^	9.14 ± 0.19^e^	9.30 ± 0.18^e^	15.30 ± 0.18^a^	11.44 ± 0.23^c^	12.30 ± 0.20^b^	< 0.0001
AST (U/L)	23th day	140.20 ± 0.45^d^	121.40 ± 0.26^e^	123.00 ± 0.77^e^	182.00 ± 0.57^a^	150.00 ± 0.41^c^	152.00 ± 0.84^b^	< 0.0001
	37th day	162.42 ± 0.59^d^	152.84 ± 1.74^e^	149.00 ± 0.51^f^	196.00 ± 0.32^a^	176.40 ± 6.12^c^	185.46 ± 2.78^b^	< 0.0001
ALP (U/L)	23th day	509.00 ± 9.00^d^	450.00 ± 3.53^e^	470.00 ± 5.24^e^	660.00 ± 9.35^a^	555.00 ± 16.58^c^	600.00 ± 18.16^b^	< 0.0001
	37th day	710.00 ± 6.51^d^	600.00 ± 11.40^e^	605.00 ± 8.66^e^	876.00 ± 8.86^a^	792.00 ± 11.57^c^	830.00 ± 14.49^b^	< 0.0001
Tp (gm/dl)	23th day	3.46 ± 0.07^ab^	3.92 ± 0.09^a^	3.94 ± 0.11^a^	2.60 ± 0.17^d^	3.10 ± 0.05^c^	3.12 ± 0.11^c^	0.001
	37th day	4.10 ± 0.03^b^	4.86 ± 0.04^a^	4.82 ± 0.03^a^	2.76 ± 0.05^d^	3.82 ± 0.05^c^	3.80 ± 0.04^c^	< 0.0001
Albumin (gm/dl)	23th day	1.98 ± 0.04^a^	2.20 ± 0.07^a^	2.20 ± 0.08^a^	0.60 ± 0.08^c^	0.82 ± 0.05^b^	0.86 ± 0.05^b^	< 0.0001
	37th day	2.20 ± 0.05^b^	2.70 ± 0.03^a^	2.68 ± 0.03^a^	1.10 ± 0.03^c^	2.40 ± 0.04^ab^	2.42 ± 0.03^ab^	< 0.0001
Globulin (gm/dl)	23th day	1.48 ± 0.13^d^	1.72 ± 0.03^c^	1.74 ± 0.08^c^	2.00 ± 0.06^b^	2.28 ± 0.09^a^	2.26 ± 0.02^a^	< 0.0001
	37th day	1.90 ± 0.04^c^	2.16 ± 0.05^b^	2.14 ± 0.05^b^	1.66 ± 0.04^d^	2.40 ± 0.04^a^	2.42 ± 0.03^a^	0.001
Urea (mg/dl)	23th day	17.68 ± 0.49^d^	15.00 ± 0.17^e^	14.30 ± 0.23^e^	22.20 ± 0.57^a^	18.80 ± 0.27^c^	20.00 ± 0.26^b^	< 0.0001
	37th day	20.80 ± 0.40^d^	18.72 ± 0.24^e^	18.00 ± 0.13^e^	24.10 ± 0.30^a^	21.68 ± 0.34^c^	22.62 ± 0.16^b^	< 0.0001
Creatinine (mg/dl)	23th day	0.80 ± 0.08^d^	0.48 ± 0.04^e^	0.52 ± 0.06^e^	1.46 ± 0.02^a^	1.02 ± 0.048^c^	1.22 ± 0.08^b^	< 0.0001
	37th day	0.96 ± 0.05^d^	0.70 ± 0.04^e^	0.72 ± 0.09^e^	1.68 ± 0.03^a^	1.20 ± 0.09^c^	1.44 ± 0.11^b^	< 0.0001
Uric acid (mg/dl)	23th day	6.78 ± 0.32^d^	5.50 ± 0.68^e^	5.20 ± 0.54^e^	9.96 ± 0.57^a^	7.80 ± 0.84^c^	8.92 ± 1.13^b^	< 0.0001
	37thday	7.34 ± 0.12^d^	6.48 ± 0.13^e^	6.36 ± 0.25^e^	9.98 ± 0.15^a^	8.14 ± 0.38^c^	8.94 ± 0.31^b^	< 0.0001

a, b, c, d, and e are means carrying different superscripts are significantly different at p < 0.05.

ALT, alanine aminotransferase; AST, aspartate aminotransferase; ALP, alkaline phosphatase; TP, total proteins.

### Effects of probiotic and prebiotic supplementation on some serum immunological parameters

Table 4 depicts that, on both the 23rd and 37th days of the experiment, a significant increase in serum IgA was recorded in chicks treated with *L. plantarum* or amylase enzyme when compared with the control group. *E. coli-*infected non-treated birds showed a significant increase in serum IgA levels in 23-day-old birds followed by a significant decrease in 37-day-old birds. Moreover, the *E. coli-*infected treated groups with *L. plantarum* or amylase enzyme showed a significant increase in serum IgA compared with the infected non-treated group over the experimental period. G6 showed the highest value on both the 23rd and 37th days of the experiment. Moreover, birds treated with *L. plantarum* or amylase enzyme showed a decrease in serum IL6 during the experimental period, and the lowest value was recorded in G3, while the birds infected with *E. coli* untreated group recorded a significant increase in serum IL6 as compared with the control group. Compared with the *E. coli-*infected non-treated group, G5 and G6 revealed a significant increase in serum IL6, and its highest value was recorded in G6. On both the 23rd and 37th days of the experiment, chickens treated with *L. plantarum* or amylase enzyme recorded a significant increase in phagocytic percent and index, and the highest value was noticed in G3 as compared with the control group (G1). Furthermore, the infected *E. coli-*non-treated chicks manifested a significant decrease in the aforementioned phagocytic parameters compared with the control group. The *E. coli-*infected treated chickens with *L. plantarum* or amylase enzyme revealed a significant increase in phagocytic percentage and index, and the highest value was observed in G6 when compared with the *E. coli-*infected non-treated group.

**Table 4:** Effect of probiotic and prebiotic supplementation on some immunological parameters in chickens of different groups on 23rd and 37th days of the experimental period (mean values ± S.E).

**Parameters**	**Period**	**Control**	***Lactobacillus plantarum***	**Amylase**	***E. coli***	***Lactobacillus plantarum*** **+ *E. coli***	**Amylase + *E. coli***	**P value**
IgA (mg/ml)	23th day	0.306 ± 0.04^e^	0.432 ± 0.02^d^	0.442 ± 0.03^d^	0.566 ± 0.05^c^	0.680 ± 0.02^b^	0.790 ± 0.02^a^	< 0.0001
	37th day	0.374 ± 0.03^e^	0.500 ± 0.02^d^	0.610 ± 0.03^c^	0.196 ± 0.04^f^	0.740 ± 0.02^b^	0.846 ± 0.03^a^	< 0.0001
IL6 (Pg/ml)	23th day	46.74 ± 0.76^d^	38.20 ± 2.55^e^	33.50 ± 1.84^f^	52.50 ± 0.83^c^	66.20 ± 1.46^b^	78.40 ± 0.88^a^	< 0.0001
	37th day	68.10 ± 0.55^d^	64.06 ± 1.12^e^	60.15 ± 1.88^f^	78.70 ± 1.04^c^	83.42 ± 1.62^b^	87.83 ± 1.17^a^	< 0.0001
Phagocytic %	23th day	72.80 ± 1.15^c^	76.00 ± 0.94^b^	79.20 ± 0.58^a^	55.80 ± 1.28^f^	65.80 ± 0.86^e^	68.80 ± 0.86^d^	< 0.0001
	37th day	73.80 ± 0.86^c^	76.40 ± 0.92^b^	80.00 ± 0.70^a^	59.20 ± 0.66^f^	66.80 ± 1.15^e^	70.20 ± 0.73^d^	< 0.0001
Phagocytic index	23th day	3.92 ± 0.10^c^	4.30 ± 0.07^b^	5.02 ± 0.08^a^	1.84 ± 0.14^f^	3.14 ± 0.09^e^	3.56 ± 0.06^d^	< 0.0001
	37th day	4.00 ± 0.12^c^	4.32 ± 0.10^b^	5.12 ± 0.08^a^	2.16 ± 0.09^f^	3.38 ± 0.10^e^	3.68 ± 0.08^d^	< 0.0001

a, b, c, d, e, and f are means carrying different superscripts are significantly different at p < 0.05.

IgA, immunoglobulin A; IL6, Interleukin 6.

### Effects of probiotic and prebiotic supplementation on some serum oxidative stress and antioxidant markers

As illustrated in Table 5, both groups treated with *L. plantarum* and amylase enzyme showed a significant decrease in serum malondialdehyde (MDA) on the 23rd and 37th days of the experiment. However, there was a significant increase in MDA in the *E. coli*-infected non-treated group as compared with the control. Compared with the *E. coli-*infected group, there was a significant decrease in MDA in the groups infected with *E. coli* and treated with *L. plantarum* and amylase enzyme over the experimental period, which was still higher than the control values. In addition, serum SOD and catalase activities revealed a significant increase in the birds treated with the *L. plantarum* and amylase enzyme, but a significant decrease was observed in the *E. coli-*non-treated infected chicks on 23rd and 37th days. However, birds infected with *E. coli* and treated with *L. plantarum* and amylase enzyme exhibited a significant increase in serum SOD and catalase activities as compared with the *E. coli-infected* non-treated birds.

**Table 5:** Effect of probiotic and prebiotic supplementation on some serum oxidative stress and antioxidant markers in chickens of different groups on 23rd and 37th days of the experimental period (mean values ± S.E).

**Parameters**	**Period**	**Control**	***Lactobacillus plantarum***	**Amylase**	***E. coli***	***Lactobacillus plantarum*** **+ *E. coli***	**Amylase + *E. coli***	**P value**
MDA (nmol/ml)	23th day	4.22 ± 0.08^c^	2.37 ± 0.02^d^	2.20 ± 0.08^d^	7.07 ± 0.11^a^	5.27 ± 0.06^b^	5.42 ± 0.32^b^	< 0.0001
	37th day	5.16 ± 0.05^c^	3.48 ± 0.15^d^	3.20 ± 0.08^d^	9.64 ± 0.12^a^	7.58 ± 0.18^b^	7.22 ± 0.11^b^	< 0.0001
SOD (U/ml)	23th day	1.76 ± 0.04^b^	2.14 ± 0.06^a^	2.22 ± 0.07^a^	0.47 ± 0.05^d^	1.15 ± 0.04^c^	1.02 ± 0.06^c^	0.001
	37th day	1.88 ± 0.05^b^	2.54 ± 0.12^a^	2.74 ± 0.06^a^	0.53 ± 0.07^d^	1.48 ± 0.11^c^	1.36 ± 0.05^c^	0.001
Catalase (ng/ml)	23th day	5.32 ± 0.06^b^	9.76 ± 0.15^a^	10.16 ± 0.49^a^	0.94 ± 0.04^d^	4.04 ± 0.14^c^	3.96 ± 0.25^c^	< 0.0001
	37th day	4.76 ± 0.08^b^	7.31 ± 0.11^a^	7.81 ± 0.27^a^	0.48 ± 0.05^d^	3.29 ± 0.33^c^	2.70 ± 0.20^c^	< 0.0001

a, b, c, and d are means carrying different superscripts are significantly different at p < 0.05.

MDA, malondialdehyde; SOD, superoxide dismutase.

### Effects of probiotic and prebiotic supplementation on DNA damage

**Figure 1:**
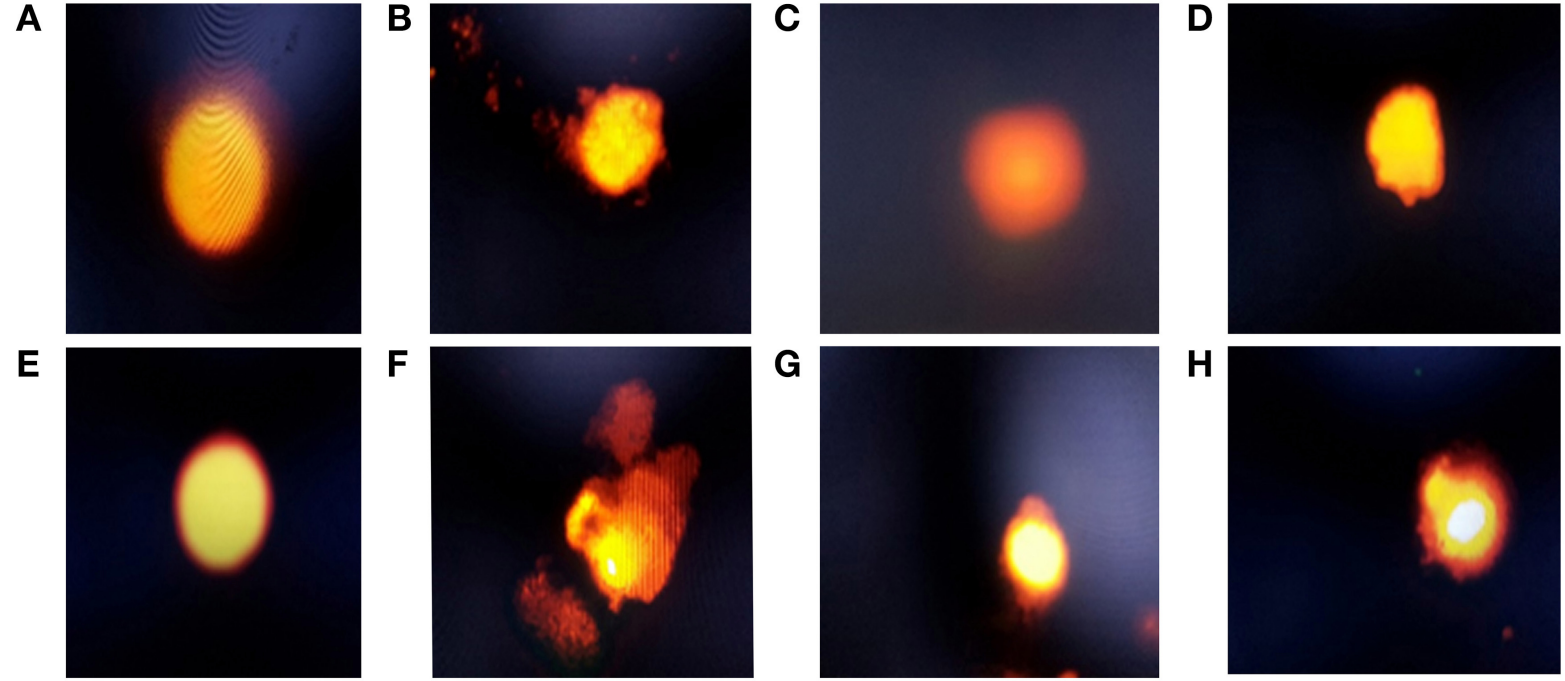
Electrophoresis of hepatic and intestinal nucleus of chickens belonging to different groups, stained with 1.2% ethidium bromide agarose (400x): **(A)** The hepatic DNA of control (G1), *Lactobacillus plantarum* (G2), and amylase enzyme (G3) groups showed no DNA damage, which was revealed by no migration of DNA fragment from the nucleus core; **(B)** The hepatic DNA of *E.coli*-infected group (G4) showed a high degree of DNA degradation, noticed by large DNA fragments migrating away from the core forming comet tail, with high nucleus core reduction; **(C)** The hepatic DNA of *E.coli*-infected group treated with *Lactobacillus plantarum* showed a minimal degree of DNA damage, with low comet tail; **(D)** The hepatic DNA of *E.coli*-infected group and treated with amylase showed a moderate degree of DNA damage with moderate comet tail; **(E)** Intestinal DNA of control, *Lactobacillus plantarum* and amylase enzyme groups showed no DNA damage; **(F)** Intestinal DNA of the *E.coli*-infected group showed a high degree of DNA damage, with large comet tail and a greatly reduced nucleus core, which showed that the DNA damage in intestinal cells was higher than the hepatic cells; **(G)** Intestinal DNA of the *E.coli*-infected group and treated with *Lactobacillus plantarum* showed a minimal degree of DNA damage with minimal comet tail, but the damage was higher than the hepatic cells; **(H)** Intestinal DNA of *E.coli*-infected group and treated with amylase enzyme showed a moderate degree of DNA damage, with moderate comet tail, which showed that these damages were higher in intestinal cells than the hepatic cells.

The results of the comet assay test in the liver and the intestine are illustrated in Figure 1 and Tables 6, 7. An improvement in the DNA degradation was observed in the group treated with the probiotic *L. plantarum*, which was represented by a decrease in comet length, tail length, head diameter, DNA percentage in the tail, tail moment, and olive tail moment with a significant increase in DNA percentage in the head, as compared to the control group. Conversely, the group treated with amylase enzyme showed a nonsignificant difference in DNA degradation throughout the experimental period when compared with the control group. The *E. coli*-infected non-treated group showed high DNA degradation and a highly significant increase in comet length, tail length, head diameter, DNA percentage in the tail, tail moment, and olive tail moment with a significant decrease in head DNA percentage as compared to the control group. Moreover, broilers infected with *E. coli* and treated with *L. plantarum* group recorded a significant decrease in DNA degradation also, and a low DNA degradation improvement was reported in the group infected with *E. coli* and treated with amylase enzyme when compared to the infected non-treated group at both the 23rd and 37th experimental days.

**Table 6:** Effect of probiotic and prebiotic supplementation on DNA damage indices in the liver tissue of chickens of different groups on 23rd and 37th days of the experimental period (mean values ± S.E).

**Parameters**	**Period**	**Control**	***Lactobacillus plantarum***	**Amylase**	***E. coli***	***Lactobacillus plantarum*** **+ *E. coli***	**Amylase + *E. coli***	**P value**
Comet length	23th day	17.28 ± 0.69^c^	14.16 ± 0.52^d^	16.78 ± 0.61^c^	24.36 ± 0.85^a^	21.50 ± 1.39^b^	23.92 ± 0.47^a^	0.007
	37th day	19.12 ± 0.26^c^	15.60 ± 0.43^d^	18.50 ± 0.59^c^	26.64 ± 0.66^a^	23.02 ± 0.84^b^	25.06 ± 0.63^a^	0.001
Head diameter (Pixel)	23th day	15.76 ± 0.75^c^	13.12 ± 0.75^d^	15.32 ± 0.71^c^	21.96 ± 0.27^a^	19.56 ± 0.33^b^	21.56 ± 0.38^a^	0.001
	37th day	17.42 ± 0.56^c^	14.40 ± 0.66^d^	16.88 ± 0.70^c^	23.88 ± 0.49^a^	20.76 ± 0.43^b^	22.38 ± 0.45^a^	0.001
DNA% in head	23th day	98.37 ± 0.17^b^	98.74 ± 0.12^a^	98.42 ± 0.17^b^	96.83 ± 0.21^d^	97.69 ± 0.20^c^	97.11 ± 0.27^d^	0.001
	37th day	98.19 ± 0.16^b^	98.74 ± 0.12^a^	98.21 ± 0.16^b^	96.60 ± 0.20^d^	97.17 ± 0.19^c^	96.65 ± 0.23 ^d^	0.001
Tail length (pixel)	23th day	1.52 ± 0.11^c^	1.04 ± 0.08^d^	1.46 ± 0.13^c^	2.40 ± 0.18^a^	1.94 ± 0.15^b^	2.36 ± 0.20^a^	< 0.0001
	37th day	1.70 ± 0.11^c^	1.20 ± 0.06^d^	1.62 ± 0.11^c^	2.76 ± 0.17^a^	2.26 ± 0.17^b^	2.68 ± 0.20^a^	< 0.0001
DNA% in tail	23th day	1.63 ± 0.10^c^	1.02 ± 0.10^d^	1.58 ± 0.07^c^	3.17 ± 0.16^a^	2.31 ± 0.16^b^	2.89 ± 0.35^a^	< 0.0001
	37th day	1.81 ± 0.11^c^	1.26 ± 0.10^d^	1.79 ± 0.13^c^	3.40 ± 0.15^a^	2.83 ± 0.09^b^	3.35 ± 0.14^a^	< 0.0001
Tail moment (arbitrary units)	23th day	0.02 ± 0.001^c^	0.01 ± 0.001^d^	0.02 ± 0.002^c^	0.05 ± 0.003^a^	0.03 ± 0.006^b^	0.04 ± 0.003^a^	0.001
	37th day	0.03 ± 0.001^c^	0.02 ± 0.002^d^	0.03 ± 0.002^c^	0.06 ± 0.002^a^	0.04 ± 0.003^b^	0.05 ± 0.005^a^	0.001
Olive tail moment	23th day	0.19 ± 0.03^c^	0.10 ± 0.02^d^	0.18 ± 0.01^c^	0.37 ± 0.02^a^	0.27 ± 0.03^b^	0.36 ± 0.03^a^	< 0.0001
	37th day	0.28 ± 0.02^c^	0.20 ± 0.02^d^	0.27 ± 0.01^c^	0.43 ± 0.02^a^	0.35 ± 0.02^b^	0.42 ± 0.03^a^	0.003

a, b, c, and d are means carrying different superscripts are significantly different at p < 0.05.

**Table 7:** Effect of probiotic and prebiotic supplementation on DNA damage indices in the intestine of chickens of different groups on 23rd and 37th days of the experimental period (mean values ± S.E).

**Parameters**	**Period**	**Control**	***Lactobacillus plantarum***	**Amylase**	***E. coli***	***Lactobacillus plantarum*** **+ *E. coli***	**Amylase + *E. coli***	***p*** **value**
Comet length	23th day	17.12 ± 0.43^c^	14.00 ± 0.42^d^	17.00 ± 0.48^c^	26.00 ± 0.56^a^	22.60 ± 0.64^b^	24.80 ± 0.60^a^	< 0.0001
	37th day	19.0 ± 0.50^c^	15.4 ± 0.30^d^	18.7 ± 0.31^c^	27.8 ± 0.46^a^	23.1 ± 0.60^b^	27.3 ± 0.52^a^	< 0.0001
Head diameter (pixel)	23th day	15.62 ± 0.79^c^	13.00 ± 0.78^d^	15.52 ± 0.75^c^	23.30 ± 0.49^a^	20.48 ± 0.48^b^	22.26 ± 0.39^a^	< 0.0001
	37th day	17.38 ± 0.29^c^	14.30 ± 0.33^d^	17.14 ± 0.26^c^	24.90 ± 0.49^a^	20.76 ± 0.30^b^	24.52 ± 0.47^a^	0.007
DNA% in head	23th day	98.40 ± 0.17^b^	99.00 ± 0.10^a^	98.43 ± 0.17^b^	96.64 ± 0.22^d^	97.30 ± 0.20^c^	96.70 ± 0.27^d^	0.009
	37th day	98.30 ± 0.11^b^	98.90 ± 0.11^a^	98.35 ± 0.12^b^	96.25 ± 0.15^d^	97.10 ± 0.19^c^	96.44 ± 0.32^d^	< 0.0001
Tail length (pixel)	23th day	1.50 ± 0.13^c^	1.00 ± 0.13^d^	1.48 ± 0.16^c^	2.70 ± 0.12^a^	2.12 ± 0.12^b^	2.54 ± 0.16^a^	< 0.0001
	37th day	1.62 ± 0.09^c^	1.10 ± 0.08^d^	1.56 ± 0.11^c^	2.90 ± 0.18^a^	2.34 ± 0.15^b^	2.78 ± 0.18^a^	< 0.0001
DNA% in tail	23th day	1.60 ± 0.13^c^	1.00 ± 0.10^d^	1.57 ± 0.15^c^	3.36 ± 0.17^a^	2.70 ± 0.11^b^	3.30 ± 0.16^a^	0.001
	37th day	1.70 ± 0.08^c^	1.10 ± 0.09^d^	1.65 ± 0.06^c^	3.75 ± 0.09^a^	2.90 ± 0.12^b^	3.56 ± 0.30^a^	< 0.0001
Tail moment (arbitrary units)	23th day	0.02 ± 0.002^c^	0.01 ± 0.001^d^	0.02 ± 0.001^c^	0.06 ± 0.002^a^	0.04 ± 0.005^b^	0.05 ± 0.003^a^	< 0.0001
	37th day	0.03 ± 0.001^c^	0.02 ± 0.005^d^	0.03 ± 0.001^c^	0.09 ± 0.001^a^	0.07 ± 0.002^b^	0.08 ± 0.001^a^	< 0.0001
Olive tail moment	23th day	0.18 ± 0.02^c^	0.10 ± 0.01^d^	0.17 ± 0.01^c^	0.40 ± 0.02^a^	0.30 ± 0.02^b^	0.40 ± 0.03^a^	0.001
	37th day	0.22 ± 0.02^c^	0.15 ± 0.01^d^	0.20 ± 0.01^c^	0.55 ± 0.01^a^	0.40 ± 0.02^b^	0.50 ± 0.02^a^	0.001

a, b, c, and d are means carrying different superscripts are significantly different at p < 0.05.

### Histopathological examination

**Figure 2:**
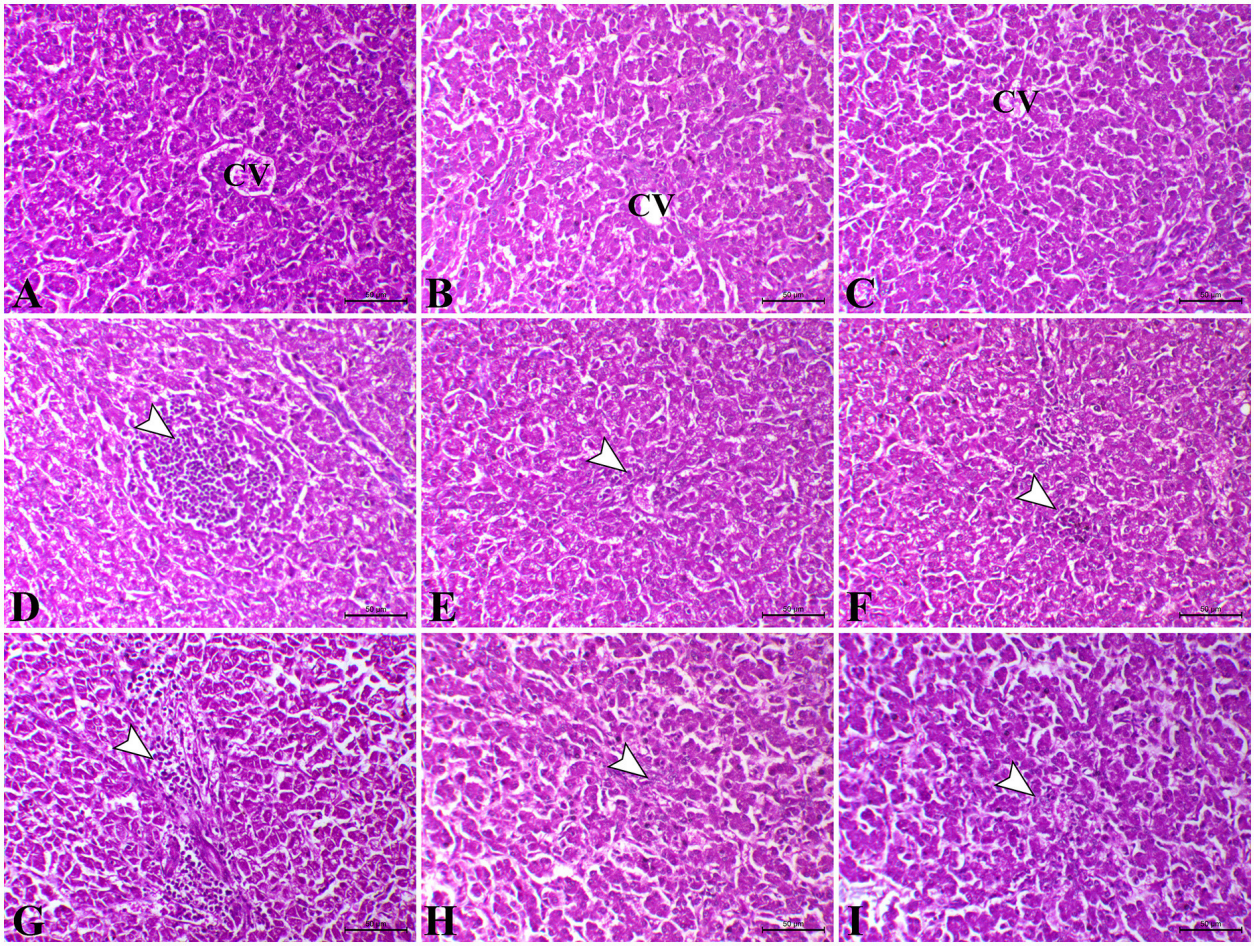
Photomicrograph of H&E stained the liver from control chicken showing normal hepatic parenchyma **(A)**. The liver of *L. plantarum* chicken showed normal tissue architecture and cellular details **(B)**. The liver of amylase-treated group showing normal hepatic parenchyma **(C)**. The liver of *E.coli*-infected chicks on the 23rd day showing focal hepatic necrosis associated with infiltration of inflammatory cells (arrowhead) **(D)**. The liver of *E.coli*-infected chicken treated with *L. plantarum* on the 23^rd^ day showing slight perivascular inflammatory cells infiltration (arrowhead) **(E)**. The liver of *E.coli*-infected chickens treated with amylase on the 23^rd^ day showing congestion of sinusoids and tiny necrotic foci (arrowhead) **(F)**. The liver of *E.coli*-infected chicken on the 37th day showing marked perivascular aggregation of inflammatory cells infiltration (arrowhead) and fibrosis **(G)**. The liver of *E.coli*-infected chicken treated with *L. plantarum* on the 37^th^ day showing congestion of hepatic sinusoids (arrowhead) **(H)**. The liver of *E.coli*-infected chicken treated with amylase on the 37th day showing mild atrophy of some hepatocytes and severe congestion of hepatic blood vessels (arrowhead) **(I)**. H&E, bar = 50 μm.

As shown in Figures 2A–C, the liver of control chicks and those treated with *L. plantarum* and amylase enzyme revealed normal tissue architecture and cellular details with no significant lesions on the 23rd and 37th days. In contrast, on the 23rd day, the liver of the *E. coli*-infected group revealed severe dilated and congestion of hepatic blood vessels (central veins and sinusoids) with focal periportal and inflammatory cells (macrophages, lymphocytes, and plasma cells predominantly) infiltration combined with severe degenerative changes as vacuolar degeneration with atrophied hepatocytes (Figure 2D). Moreover, on the 23rd day, the liver of *E. coli-*infected chicks administered with *L. plantarum* revealed normal hepatic parenchyma with dilated hepatic sinusoids, mild perivascular inflammatory cells infiltration with congestion of hepatic sinusoids, and mild degeneration in hepatocytes (Figure 2E). In addition, *E. coli-*infected chicken treated with amylase enzyme exhibited slight congestion of both hepatic blood vessels and sinusoids on the 23rd day. Some chickens of the same group exhibited perivascular infiltration of leucocytic cells and von Kupffer cells (Figure 2F). While, on the 37th day, the liver sections of *E. coli-*infected chicks showed a disappearance of basic architecture, in addition to multifocal coagulative necrosis represented in the karyoltic nuclei of hepatocytes, perivascular aggregation of leucocytic cells and fibroblast, and hyperemia (Figure 2G). On the 37th day, in most cases, the liver of the *E. coli-*infected chicks administered with *L. plantarum* restored their normal histomorphologic picture of tissue architecture and cellular details, while some cases demonstrated mild diffuse atrophied hepatocytes and congestion of both hepatic blood vessels and hepatic sinusoids (Figure 2H), while, on the 37th day, the liver sections of *E. coli-*infected chicken treated with amylase enzyme showed normal tissue architecture and cellular details, and mild diffuse coagulative necrosis is represented in karyolysis of hepatic cells nuclei with slight lymphocytic cells infiltration (Figure 2I).

**Figure 3:**
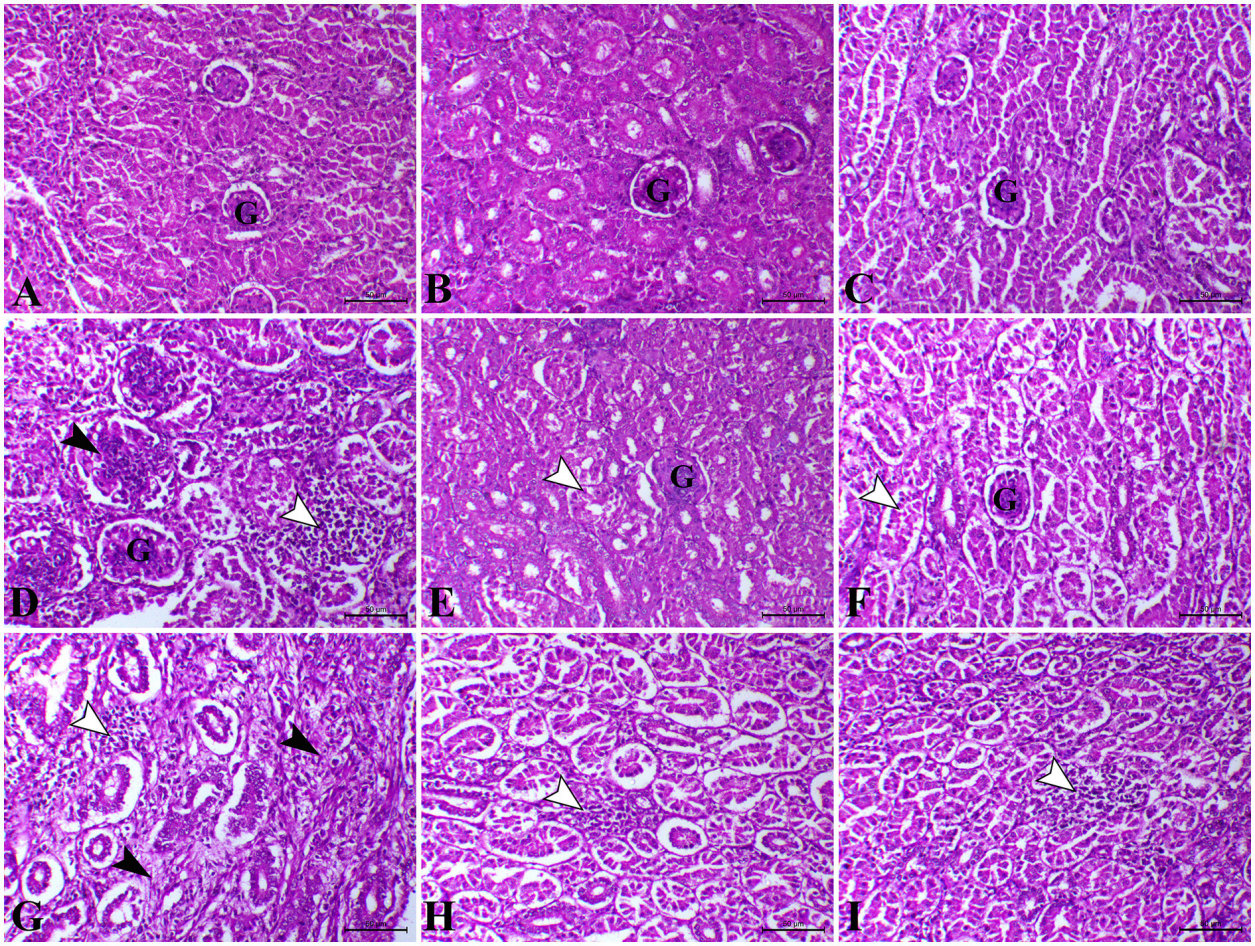
Photomicrograph of H&E stained kidney from control chicken shows the following: **(A)** Normal renal glomeruli and tubules; **(B)** The kidney of *L. plantarum* chicks showing normal renal glomeruli and tubules; **(C)** The kidney of amylase-treated chicken showing apparently normal renal glomeruli and tubules; **(D)** The kidney of *E.coli*-infected chicks at 23rd day showing marked interstitial nephritis (white arrowhead) associated with proliferative glomerulonephritis (black arrow);. **(E)** The kidney of *E.coli*-infected chicks treated with *L. plantarum* at 23rd day showing decreased tubular degeneration (arrowhead); **(F)** The kidney of *E.coli*-infected chicks treated with amylase at 23rd day showing a moderate degree of tubular degeneration (arrowhead); **(G)** The kidney of *E.coli*-infected chicks at 37th day showing interstitial nephritis associated with focal aggregation of leucocytic cells (white arrowheads) and interstitial fibrosis (black arrowheads); **(H)** The kidney of *E.coli*-infected chicks treated with *L. plantarum* at 37th day showing a mild degree of interstitial nephritis (arrowhead); **(I)** The kidney of *E.coli*-infected chicks treated with amylase at 37th day showing a mild degree of interstitial nephritis (arrowhead). H&E, bar = 50 μm.

As shown in Figures 3A–C, on the 23rd and 37th days, the kidney of control, *L. plantarum*, and amylase enzyme chicks showed normal renal cortex and medulla. On the 23rd day, the kidney of *E. coli-*infected chicks revealed severe congestion of renal blood vessels with interstitial hemorrhage besides degenerative changes in the epithelial lining of the renal tubule (Figure 3D). Moreover, intrarenal hyaline casts depositing with degeneration of some renal epithelium were also detected in the kidney of *E. coli* infected chicks. Furthermore, on the 23rd day, the kidney sections of *E. coli-*infected chicks pre-treated with *L. plantarum* showed apparently normal renal cortex and medulla with mild focal intertubular hemorrhage (Figure 3E). Also, on the 23rd day, infected-*E.coli* chicken after amylase enzyme treatment showed normal renal cortex and medulla with cystic dilation of some renal tubules and moderate congestion of blood vessels (Figure 3F). While on the 37th day, the kidney sections of the *E. coli* infected group showed massive aggregation of leucocytic cells besides focal necrosis of renal tubules and congestion of renal blood vessels (Figure 3G). On the 37th day, the kidney sections *E. coli-*infected chicks pre-treated with *L. plantarum* that belong to the same group showed a reduction in renal lesions with few lesions still as degenerations and atrophy of some renal tubules (Figure 3H). While, on the 37th day, the kidney sections of infected-*E. coli* chicken after amylase enzyme treatment showed improvement of lesions with mild diffuse intertubular hemorrhage and mild congestion combined with degeneration of some renal tubules with hypercellularity of some renal glomeruli (Figure 3I).

**Figure 4:**
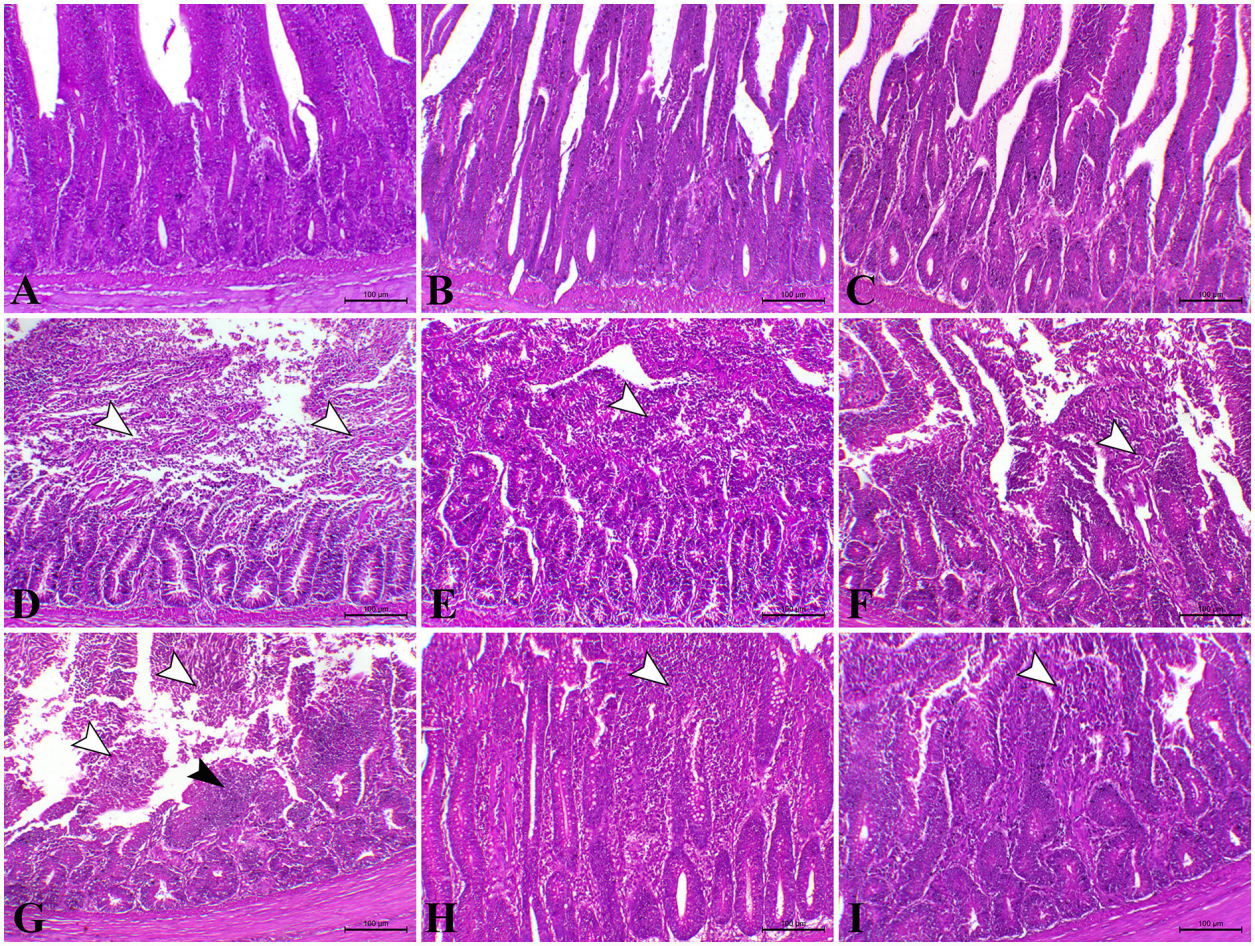
Photomicrograph of H&E stained intestinal section of control chicken shows the following: **(A)** Normal mucosal lining; **(B)** The intestine of chicken treated with *L. plantarum* showing normal mucosa and submucosa; **(C)** The intestine of chicken treated with amylase showing normal mucosa and submucosa; **(D)** The intestine of *E.coli*-infected chicken at 23rd day showing severe degree of necrotic enteritis (arrowheads); **(E)** The intestine of *E.coli*-infected chickens treated with *L. plantarum* at 23rd day showing decreased necrotic enteritis lesions (arrowhead); **(F)** the intestine of *E.coli*-infected chickens treated with amylase at 23rd day showing a moderate degree of necrotic enteritis (arrowhead) **(G)** The intestine of *E.coli*-infected infected chickens at 37th day showing necrotic enteritis associated with atrophy of intestinal villi (white arrowhead) with regenerative attempts of mucosa (black arrowhead); **(H)** The intestine of *E.coli*-infected chickens treated with *L. plantarum* at 37th day showing mild catarrhal enteritis (arrowhead); **(I)** The intestine of *E.coli*-infected chicken treated with amylase at 37th day showing a moderate degree of catarrhal enteritis (arrowhead).

As illustrated in Figures 4A,B, the intestine of control and *L. Plantarum* chicks on the 23rd and 37th days recorded normal mucosa and submucosa. Furthermore, chicks treated with the amylase enzyme, at the 23rd and 37th days, recorded normal mucosa and submucosal with elongated villi (Figure 4C). In contrast, the intestine of the *E. coli-*infected group, on the 23rd day, showed vacuolation of submucosal glandular epithelium besides edema among muscle fiber of muscularis mucosa (Figure 4D). However, on the 23rd day, the intestinal sections of *E. coli-*infected chicks treated with *L. plantarum* showed normal mucosa and submucosa combined with congestion of serosa blood vessels (Figure 4E). Diffuse metaplasia of intestinal enterocytes to goblet cells (mucinous degeneration) was noticed in the same later group. Also, the intestinal sections of *E. coli-*infected chicks administered with amylase enzyme showed elongated villi with or without mild mucinous degeneration (Figure 4F) on the 23rd day besides mild focal submucosal glandular atrophy and leucocytic cells infiltration. While, on the 37th day, the intestinal sections revealed severe inter-glandular and lamina propria inflammatory cells infiltration, mainly lymphocytes, besides atrophy of some intestinal glands (Figure 4G), and atrophy of some submucosal glands was also seen in some cases of *E. coli-*infected chicks. On the 37th day, the intestinal sections of some chicks belonging to *E. coli-*infected chicks treated with *L. plantarum* group exhibited normal intestinal layers with a fusion of some intestinal villi (Figure 4H). However, on the 37th day, the intestinal sections belonging to *E. coli-*infected chicks administered with amylase enzyme group showed apparently normal mucosal structure with focal infiltration of inflammatory cells in lamina propria (Figure 4I).

## Discussion

To ensure food free of drugs for consumers (41), feed additives have been used as alternatives to in-feed antibiotics to prevent the risk of developing pathogens. Probiotics and prebiotics are used as growth promoters, can be used as non-antibiotic feed additives substitute, and enhance the growth performance of broiler chickens. The present study revealed a series of interesting findings in relation to the modulatory effect of dietary probiotic and prebiotic supplementations on growth, immuno-biochemical alteration, DNA damage, and pathological changes in *E. coli-*infected broiler chicks, which was revealed in this present study. Consistent with several previous reports (42, 43), supplementation of probiotics (*L. plantarum*) and prebiotics (amylase enzyme) in diet improved the body performance parameters (BW, BWG, and FI) and decreased the FCR. Weight gain in the probiotic-treated group (G2 and G5) might result from preserving healthy intestinal flora by competitive exclusion and antagonism, increasing digestive enzyme activities, and encouraging the digestion rate of energy nutrients (44). Moreover, treatment of infected birds (G6) with exogenous enzymes (amylase enzyme) might have both direct and indirect actions by providing a suitable environment for the endogenous digestive enzymes to act on the substrate, adjustment of the intestinal microbial populations, speed feed passage rate by increasing the hydrolytic GIT capacity and increasing the availability of macronutrients that are resistant to digestion by endogenous enzymes, leading to enhancement of nutrient solubility, digestibility and availability [40]. The growth performance of *E. coli*-infected non-treated chicks (G4) revealed a significantly lower BW, BWG, and FI besides an increase in FCR in comparison with control chicks. These present results were consistent with those previously obtained by Liang et al. (45) and Wu et al. (46), who noticed a reduction of FI and growth retardation due to intestinal lesions resulting from the challenges with *E. coli*, which was associated with villous atrophy and intestinal morphology disorder and consequently reduced nutrients absorption and/or hepatic lesions or kidney dysfunction (42, 47). In addition, the groups infected with *E. coli* and treated with *L. plantarum* and amylase enzyme (G5&G6) showed an improvement in body performance and reduction in the FCR as compared to the infected non-treated group. These findings are in agreement with previous studies (48).

It is noteworthy to mention that the liver enzymes (ALT, AST, and ALP) are among the most common indicators of hepatic functions. Our result revealed a significant decrease in ALT, AST, and ALP in treated groups with *L. plantarum* and amylase enzyme (G2&G3) than in control, which may be attributed to the hepatoprotective effect associated with different xenobiotics (49–51). A significant increase in the liver enzymes was noticed in *E. coli-*infected non-treated birds all over the experimental period, which may be attributed to hepatocellular damage during the detoxification of *E. coli* and bacterial toxins and may have led to hepatocyte membrane disruption associated with leakage of hepatocyte contents and, therefore, the targeted elevation of the serum hepatic enzymes (52). On the contrary, the *E. coli-infected* groups treated with *L. plantarum* and amylase enzyme (G5 and G6) had a significant decrease in the liver enzymes than the infected non-treated group. The hepatoprotective effect of both probiotics and prebiotics may be due to both antimicrobial and antioxidant effects and enhanced immune status that decreased the pathogenic bacterial population (53). Regarding the renal function parameters, serum creatinine, urea, and uric acid were assessed, and the present study revealed a significant decrease in serum creatinine, urea, and uric acid in G2 and G3 in all experimental periods, which were consistent with several previous reports (54, 55). However, other studies (56, 57) reported that the addition of either probiotics or prebiotics had no significant change in renal enzymes. This contradiction could be attributed to the type, number, and strain of bacteria present in probiotics and the types of exogenous enzymes used in the prebiotic. There is an increase in serum renal function parameters in the *E. coli-infected* non-treated group compared with the other groups (58), which may be attributed to renal damage, imbalance of protein metabolism, and imbalance of amino acids concentration (53). Both G5 and G6 showed improvement of renal functional tests that may be explained through decreasing urea synthesis, and the improvement of protein metabolism balance (59).

The present study revealed that *L. plantarum* and amylase enzyme-treated birds demonstrated a significant increase in total protein serum level, albumin, and total globulins when compared with the control group. Probiotics were reported to be associated with an increased anabolic than the catabolic process by increasing the absorptive capacity of the intestine due to histomorphological changes and higher intestinal enzyme activity (60–63). In addition, probiotics increased total globulins, which play a great role in the host's immune system (5). Conversely, the *E. coli-infected* non-treated birds (G4) showed a significant decrease in serum total protein and albumin over the experiment period as compared with the control group. The total globulins in this group showed a significant increase on the 23rd day and then was followed by a significant decrease on the 37th day. Data were supported by the previously obtained results (64). Damage to the intestinal barrier encourages systemic bacterial population, which in turn results in the liver damage, intestinal malabsorption, and kidney dysfunction associated with excessive degradation of plasma protein by bacterial endotoxins (52). The initial increase of total globulin could be associated with antigenic stimulation of the infecting microorganism, while the later decrease of globulin levels was accompanied by the liver damage (56). Serum IgA is considered one of the most important humoral factors produced by B-cells. Therefore, this acts as the first line of defense against infection (65). Our data revealed a significant increase in serum IgA levels in G2 and G3 as compared with the control, which is consistent with several previous studies (57, 66). The *E. coli-infected* non-treated birds (G4) revealed an initial increase in serum IgA levels on the 23rd day, which was then decreased on the s37th day. There is some evidence that infection stimulates the immune system to a limited extent, leading to an increase in serum IgA. With time, the infection suppresses the immune system, leading to a decreased serum IgA (46, 67). The infected treated birds (G5 and G6) showed enhanced serum IgA levels as compared to G4. This indicates that the use of probiotics and prebiotics might stimulate the intestinal mucosa to produce more IgA after the *E. coli* challenge, which blocks the receptors and diminishes the number of pathogenic bacteria in the intestinal lumen (42).

Importantly, cytokines are immunoregulatory peptides that contribute to innate and adaptive immunity, and therefore, they play an essential role in immunoregulation (68). IL-6 is a pleiotropic cytokine produced by T cells and macrophages. This cytokine simulates the immune response, e.g., during infection and any tissue damage causing inflammation, and has proinflammatory and anti-inflammatory roles, which are critical in fighting infection (69).

In the present study, birds treated with *L. plantarum* and amylase enzyme (G2 and G3) showed a significant decrease in IL6 compared to chickens fed with normal ration. Similarly, a previous study (70) indicated that probiotic supplementation in a piglet diet led to decreased proinflammatory cytokines. In contrast, chicken challenged with *E. coli* (G4) showed a significant increase in serum IL6, which is consistent with a previous study (71), and the possible explanation could be due to the infection with bacteria, as *E. coli* enhanced production of extreme levels of IL6 (72, 73). It should be noted that *E. coli* has molecular patterns on its surface, such as lipopolysaccharides (LPS) and flagellin that bind with toll-like receptors on macrophage surfaces, causing interleukins to be secreted (IL-1, IL-6, and IL-8) (74, 75). Meanwhile, the infected groups supplemented with probiotic and prebiotic (G5 and G6) showed a significant improvement in serum IL6 compared with G4. Present data are supported by previous studies (65, 76), recording the intake of probiotics by *E. coli*-infected birds which enhanced the production of proinflammatory cytokines such as IL-1, TNF-α, and IL-6. This study also showed a significant increase in phagocytic percentage and phagocytic index in G2 and G3 on the 23rd and 37th days. These findings are in line with a previous study (57) that found dietary supplementation with both multi-enzyme or probiotic preparations either singly or in combination could enhance phagocytic activity and phagocytic index of broiler chicks than control. On the contrary, the control positive group (G4) birds showed a significant decrease in phagocytic percentage and phagocytic index at the 23rd and 37th days of age compared with the control negative group. These findings were consistent with previous study (77), which attributed these findings to bacterial endotoxins that caused pathophysiological effects and induced suppression of antibacterial defense mechanism or/and may be due to exhaustion of immune system by *E. coli* infection (68). The *E. coli-*infected birds treated with *L. plantarum* and amylase enzyme were significantly higher in phagocytic activity and phagocytic index as compared with the *E. coli-*infected non-treated birds. The improvement in phagocytic activity indicated that probiotics or prebiotics directly promote maturation and activation of macrophages (55).

Malondialdehyde is considered one of the markers for oxidative damage of the lipid peroxidation level (78). Compared to normal control birds, the serum MDA levels significantly decreased in probiotic and prebiotic treated groups (G2 and G3), which may be attributed to the positive modulation of the dynamics of oxidants and antioxidants in the body of chickens. It seems that the enhancement of the intestinal flora would selectively improve the ability to chelate free radicals, capturing reactive oxygen species (ROS) and inhibiting their cytotoxic activities (79). Similar results were previously reported by Wu et al. (51) and Saleh et al. (43). Consistent with results obtained in a previous study (71, 80), serum MDA significantly increased in *E. coli-infected* chickens (G4). This increase in the serum MDA may be attributed to the *E. coli* endotoxins, which induced extensive damage to a variety of organs, including the liver, overproduction of ROS, and reactive nitrogen species targeting oxidative stress through cell damage as it can attack protein and nucleic acid of the cell. MDA can also attack the polyunsaturated fatty acids of the membrane lipids, which in turn trigger lipid peroxidation and increase the activity of antioxidant enzymes to minimize the effect of ROS by the production of antioxidants (55). G5 and G6 infected with *E. coli* and treated with *L. plantarum* and amylase enzyme showed a significant decrease in serum MDA levels compared with the infected group. This observation could be attributed to the antioxidant effect of the supplemented probiotic and prebiotic that confer sufficient protection against lipid peroxidation, increased glutathione concentrations, and reduced intestinal oxidation (8, 55).

Serum SOD and CAT enzymes play a critical role in the protection of the cell from oxidative damage by ROS. SOD is an enzyme found in all living cells, and it degrades the potentially harmful oxygen molecules in cells through the decomposition of hydrogen peroxide into water and oxygen. This present study showed an increase in serum SOD and catalase activity in probiotic- and prebiotic-treated chickens, suggesting the modulated dynamics of oxidants and antioxidants by improved gut microbes and that microbes, in turn, released some bioactive substances that could potentially prevent oxidative damage (81). The same results were recorded by He et al. (82) and Wang et al. (66). On the other hand, *E. coli*-infected non-treated chickens (G4) showed a significant decrease in serum SOD and catalase activity, which is nearly similar to the results that were obtained (64). The infection decreased the antioxidant defense system, which could be compensated by the cellular defense systems, or these reactive compounds may inhibit the enzyme activity, leading to a significant decrease in SOD and catalase activities, which catalyze the dismutation of the superoxide anion into hydrogen peroxide. Molecular oxygen is considered one of the most potent antioxidant. As mentioned above, catalase is a widely distributed enzyme that destroys hydrogen peroxide ROS, which is a toxic product of both normal aerobic metabolism and pathogenic ROS production (81). In the present study, the groups infected by *E. coli* and treated with *L. plantarum* and amylase enzyme (G5 and G6) showed a significant elevation in serum SOD and catalase activity in comparison with the control positive group (G4). Our present findings are partially in agreement with the results of Dong et al. (83), who illustrated that probiotics could enhance the activity of SOD in chickens infected with *E. coli*, and treated with probiotic than infected non-treated chickens. It is therefore not surprising to state that the addition of probiotics and prebiotics might play a critical role in the recovery of intestinal linings that are in continuous interaction with the microorganisms and inhibit excess oxidative free radicals that may cause cell damage (84).

From the available literature studies, comet assay is widely recognized as a sensitive technique for studying DNA damage and repair. In this assay, cells with high DNA damage show the migration of chromosomal DNA from the nucleus toward the anode like the shape of a comet. The results of the current study indicated that dietary *L. plantarum* supplementation reduced DNA damage in the liver and the intestinal tissues than the control. The obtained data are in keeping with some previous reports (85, 86), recording that probiotics can reduce DNA damage caused by any endogenous or external source of stress. The possible explanation was attributed to the role of probiotics in the detoxification and elimination of chemicals and heavy metal toxicity in the body (87). Probiotics act as free radical scavengers and antioxidants by decreasing the oxidative stress that causes DNA damage and genotoxicity, which DNA fragmentations in the comet assay, leading to excision and repair of oxidized bases (88). The group treated with prebiotic (G3) showed non-significant difference in DNA degradation at the 23rd and 37th days of age compared with the control group. These results were in stark contrast with those obtained (89, 90). In contrast, the *E. coli-infected* non-treated group (G4) showed high DNA damage in the intestine and the liver, which was consistent with several previous reports, revealing that *E. coli* strain induced DNA double-strand breaks and chromosomal abnormalities in eukaryotic cells (80, 91, 92). Another study (93) reported that the endotoxin shock induced by *E. coli* infection was responsible for the overexpression of apoptosis-related genes increasing DNA damage to the liver and the brain cells of laying hens. This may result from the activation of the caspase-9- and caspase-3-dependent branch of the apoptotic pathway in cell line during the uptake and digestion of *E. coli* bacteria. Likewise, the proteolytic action of the caspase family on specific cell substrates led to apoptosis including DNA damage (94). In addition, we should note that oxidative stress induced by *E. coli* infection induced high ROS production, which resulted in DNA strand breaks (80). The infected birds treated with probiotic were more effective in genoprotective effect against DNA damage compared with G4 in the present study. This concept that *L. plantarum* supplementation improved DNA damage caused by *E. coli* infection in broilers was supported by the findings of a previous study (6). The present findings are consistent with the hypothesis that suggests probiotics increase intestinal acidity, which is conducive to reducing the pathogen population in the gut of chickens, and therefore, leading to the reduction of DNA damage caused by infection (95). However, we should note that a partial genoprotective effect against DNA damage was observed in infected birds treated with amylase enzyme compared with G4. The limited genoprotective role of the addition of exogenous enzymes in this study is attributed to the insufficiency of enzyme activity and dosage used. Our findings in this study revealed severe alteration of hepatic, renal, and intestinal tissues in the *E. coli-*infected non-treated group (G4). The hepatic tissue of this group showed multiple necrotic foci associated with the aggregation of mononuclear cells. Inflammation was extended to renal tissues associated with interstitial nephritis. In addition, the main lesion was noticed as severe necrotic enteritis. This lesion was consistent with several previous works (64, 96). Interestingly, using *L. plantarum* and amylase enzyme as a prophylactic against *E. coli* revealed a marked decrease in the inflammatory lesions within the liver, the kidney, and the intestine which is consistent with several previous reports (44, 97).

## Conclusion

This study concluded that the experimental infection with *E. coli* strain O78 in chicks caused severe alterations in the body performance, biochemical, immunological, antioxidant, oxidative stress parameters, and histological structures with extensive DNA degeneration. Interestingly, dietary supplementation with probiotics and prebiotics improved the efficiency of poultry production with body performance and increased immune response in non-infected bird groups. Given the above information, probiotics and prebiotics might have a promising effect as prophylactic supplementation for controlling *E. coli* infection through the improvement of growth performance and returning the aforementioned parameters to near normal values with histopathological change subsidence and DNA genotoxicity.

## Data availability statement

The original contributions presented in the study are included in the article/supplementary material, further inquiries can be directed to the corresponding author.

## Ethics statement

The animal study was reviewed and approved by the Institutional Review Board of the faculty of Veterinary Medicine (Local Ethical Approval), Zagazig University, Egypt. The Approval number of the study is ZU-IACUC/2/F/88/2021.

## Author contributions

MH, AH, HA-E, WA, and EE conceived the idea and performed the methodology, formal analysis, data curation, and supervision besides revision of the manuscript. WA, ND, AA, and EE participated in the formal analysis, data curation, and contributed their scientific advice. MH, AH, HA-E, WA, ND, AA, and EE drafted the manuscript and prepared the manuscript for publication and revision. All authors have read and agreed to the published version of the manuscript.

## Funding

This work was supported by the Faculty of Veterinary Medicine, Zagazig University, Egypt.

## Conflict of interest

The authors declare that the research was conducted in the absence of any commercial or financial relationships that could be construed as a potential conflict of interest.

## Publisher's note

All claims expressed in this article are solely those of the authors and do not necessarily represent those of their affiliated organizations, or those of the publisher, the editors and the reviewers. Any product that may be evaluated in this article, or claim that may be made by its manufacturer, is not guaranteed or endorsed by the publisher.
